# Andrographolide Ameliorates Diabetic Cardiomyopathy in Mice by Blockage of Oxidative Damage and NF-*κ*B-Mediated Inflammation

**DOI:** 10.1155/2018/9086747

**Published:** 2018-06-25

**Authors:** Ershun Liang, Xue Liu, Zhanhui Du, Ruixue Yang, Yuxia Zhao

**Affiliations:** ^1^The Key Laboratory of Cardiovascular Remodeling and Function Research, Chinese Ministry of Education and Chinese Ministry of Public Health, Qilu Hospital, Shandong University, Jinan, Shandong 250012, China; ^2^Department of Cardiology, Qilu Hospital, Shandong University, Jinan, Shandong 250012, China; ^3^Department of Traditional Chinese Medicine, Qilu Hospital, Shandong University, Jinan, Shandong 250012, China

## Abstract

Andrographolide (Andro), a major bioactive component obtained from *Andrographis paniculata* Nees, has exerted wide antioxidant as well as cytoprotective properties. However, whether Andro treatment could retard the progress of diabetic cardiomyopathy (DCM) remains unknown. In this study, we evaluated the effects of Andro against diabetes-induced myocardial dysfunction and explored the underlying mechanism in STZ-induced diabetic mice. As a result, treatment with Andro dose dependently suppressed cardiac inflammation and oxidative stress, accompanied by decreasing cardiac apoptosis, which subsequently ameliorated cardiac fibrosis and cardiac hypertrophy. Further, Andro blocked hyperglycemia-triggered reactive oxygen species (ROS) generation by suppressing NADPH oxidase (NOX) activation and augmenting nuclear factor erythroid 2-related factor 2 (Nrf2) expression both *in vitro* and *in vivo*. Our results suggest that the cardioprotective effects afforded by Andro treatment involve the modulation of NOX/Nrf2-mediated oxidative stress and NF-*κ*B-mediated inflammation. The present study unravels the therapeutic potential of Andro in the treatment of DCM by attenuating oxidative stress, inflammation, and apoptosis.

## 1. Introduction

Diabetic cardiomyopathy (DCM) is characterized by myocardial left ventricular dysfunction, accompanied by the development of cardiac fibrosis, cardiac hypertrophy, and cardiomyocyte apoptosis [1]. Chronic hyperglycaemia contributes to myocardial injury and fibrosis, in the absence of underlying coronary artery disease and systemic hypertension, which subsequently induces heart failure [2, 3]. Unfortunately, there are currently no effective approaches to protect against or halt DCM in the clinic. Intensive glycemic control can only slow down but not reverse the progression of heart failure [4]. DCM is still the leading cause of morbidity and mortality in diabetics. It is urgent to elucidate the molecular mechanisms and identify novel potential compounds for the prevention and treatment of DCM.

Studies have proposed many complicated molecular events which lead to the development of DCM, including elevated oxidative damage [5, 6], cardiac inflammation [5, 6], mitochondrial dysfunction [7], cardiomyocyte apoptosis [6, 8], and cardiomyocyte hypertrophy [1]. Among all of these events, hyperglycemia-induced oxidative damage and inflammatory responses may be the upstream of signaling cascades [9, 10]. Hyperglycemia directly augments the expression of ROS-generating NADPH oxidases (including NOX2, NOX4, and p47phox) in the diabetic myocardium [11]. In addition, hyperglycemia induces the secretion of cytokines in cardiac cells via multiple pathways that converge towards nuclear factor-*κ*B (NF-*κ*B) signaling [12]. Notably, hyperglycemia-induced oxidative stress is also closely linked with NF-*κ*B signaling-mediated inflammatory responses [12]. Considering the complex crosstalk of these two events, a therapeutic agent with both antioxidant and anti-inflammatory activities is promising for the treatment of DCM [13].

Andrographolide (Andro) is one of the major bioactive components from a Chinese herbal medicine, *Andrographis paniculata* Nees [14]. Reports have shown that Andro exhibits a variety of biological activities, such as hepatoprotective [14], anti-inflammatory [15], antiviral [16], antitumor [17], antihyperglycemic [18, 19], and antioxidant properties [20]. Recently, a study by Ji et al. [21] reported that Andro treatment markedly attenuated ROS production and proinflammatory cytokines in the diabetic kidney. Yu et al. [22] found that Andro treatment could ameliorate diabetic retinopathy via attenuating retinal angiogenesis and inflammation. Although the antioxidant property of Andro has been reported in several cell types [14, 23, 24], its role in cardiomyoblasts especially in DCM remains unknown. In this study, we investigated the potential protective effects of Andro in the diabetic myocardium and H9c2 cardiomyoblasts exposed to high glucose. Our findings underscore the potential use of Andro for the prevention of DCM.

## 2. Materials and Methods

### 2.1. Animals and Experimental Protocols

Andro was a gift from Tasly Pharmaceutical Company (Tianjin, China). All animal protocols were approved by the ethics committee of Shandong University. Eight-week-old (25–30 g) C57/BL6J mice were employed, and diabetes was induced by intraperitoneal injection of streptozotocin (STZ; Sigma-Aldrich; 50 mg/kg) for five consecutive days [25]. Mice with blood glucose > 16 mmol/L were considered to be diabetic. Then diabetic mice were treated with Andro (1, 10, or 20 mg/kg/day) [26] or vehicle by intragastric gavage for consecutive 12 weeks [27]. The control mice were treated with either vehicle or Andro (20 mg/kg/day) alone for the duration of treatment.

### 2.2. Echocardiography

Cardiac function including left ventricular ejection fraction (LVEF), fractional shortening (FS), and the ratio of early to late mitral inflow velocity (E/A) was measured as previously described [8] using the Vevo770 imaging system (VisualSonics, Toronto).

### 2.3. Determination of SOD, MDA, 4-HNE, and Reactive Oxygen Species

We evaluated the levels of myocardial malondialdehyde (MDA), 4-hydroxynonenal (4-HNE), and superoxide dismutase (SOD) using commercially available kits (Jiancheng Bioengineering Institute, Nanjing, China), according to the manufacturer's instructions. We measured superoxide (O_2_^−^) levels in fresh myocardial tissues after incubation with dihydroethidium (DHE).

### 2.4. Histology, Immunohistochemistry, and TUNEL Staining

Mouse hearts were dissected and fixed in paraformaldehyde. Tissues were paraffin-embedded and sectioned (5 *μ*m). For immunohistochemistry, primary antibodies against 3-NT (1 : 100, N5538, Sigma), IL-6 (1 : 100, ab7737, Abcam), VCAM-1 (1 : 100, ab134047, Abcam), and ICAM-1 (1 : 100, ab179707, Abcam) were used. To examine extracellular matrix (ECM) deposition, sections were dewaxed and stained with Masson's trichrome or Sirius Red. To detect DNA fragmentation in myocardial tissues, TUNEL staining was performed using the In Situ Cell Death Detection Kit (Roche Applied Science, USA).

### 2.5. Reverse Transcription and Real-Time PCR

Total RNA was extracted from myocardial tissues or cells using TRIzol reagent (Invitrogen, USA) and reverse-transcribed into cDNA. Quantitative RT-PCR was used to determine the mRNA expression of target genes. The 2^−ΔΔCT^ method was employed to calculate the relative fold changes.

### 2.6. Western Blot Analysis

Total proteins were separated by SDS-PAGE, transferred on to PVDF membranes, and incubated with the following primary antibodies: anti-iNOS (1 : 1000, ab178945, Abcam), anti-p65NF-*Κ*b (1 : 1000, 8242S, Cell Signaling Technology), anti-I*κ*B*α* (1 : 1000, 4812S, Cell Signaling Technology), anti-p-I*κ*B*α* (1 : 1000, 2859S, Cell Signaling Technology), anti-Histone (1 : 1000, 4499S, Cell Signaling Technology), anti-VCAM-1(1 : 1000, ab134047, Abcam), anti-ICAM-1 (1 : 1000, ab119871, Abcam), anti-COX-2 (1 : 1000, 12282S, Cell Signaling Technology), anti-cleaved PARP (1 : 1000, 5625S, Cell Signaling Technology), anti-cleaved caspase-3 (1 : 1000, 9661S, Cell Signaling Technology), anti-Bax(1 : 1000, 2772S, Cell Signaling Technology), anti-Bcl2(1 : 1000, 3498S, Cell Signaling Technology), anti-Akt (1 : 1000, 4691S, Cell Signaling Technology), anti-phospho-Akt (p-AKT) (1 : 1000, 4060S, Cell Signaling Technology), anti-Nrf2 (1 : 1000, 12721S, Cell Signaling Technology), anti-HO-1 (1 : 1000, 70081S, Cell Signaling Technology), and anti-*β*-actin (1 : 5000, A1978, Sigma). After further incubation with corresponding secondary antibodies, signals were detected with a chemiluminescent reagent.

### 2.7. Electrophoretic Mobility Shift Assays (EMSA)

Nuclear proteins were extracted from the myocardial tissues treated with or without Andro. The biotin-labelled NF-*κ*B oligonucleotide probe used in EMSA was 5′-AGTTGAGGGGACTTTCCCAGGC-3′.

### 2.8. Cell Culture and Flow Cytometry

H9c2 cardiomyocytes were obtained from ATCC (USA). For *in vitro* experiments, cells were exposed to various concentrations of glucose and Andro. We used 2,7-dichlorofluorescein diacetate (DCFH-DA) and DHE to determine the levels of intracellular ROS in cultured H9c2 cardiomyocytes by a confocal microscopy, as previously described [8, 28]. The relative fluorescence intensity was acquired. N-acetyl-L-cysteine (NAC) (5 mM) was used as positive control.

After stimulation with high glucose, H9c2 cardiomyoblasts were collected, followed by incubation with Annexin V-FITC and propidium iodide (PI). Then the apoptosis was measured by flow cytometry (BD FACSCalibur, USA).

### 2.9. Statistical Analysis

Data are expressed as mean ± SD. Kolmogorov-Smirnov test was carried out to determine the normality of distribution [29]. Statistical comparisons were carried out by one-way ANOVA. The Student–Newman–Keuls post hoc test was used to make pairwise comparisons between means. *P* < 0.05 was considered statistically significant.

## 3. Results

### 3.1. Andro Attenuates Diabetes-Induced Myocardial Dysfunction In Vivo

**Figure 1:**
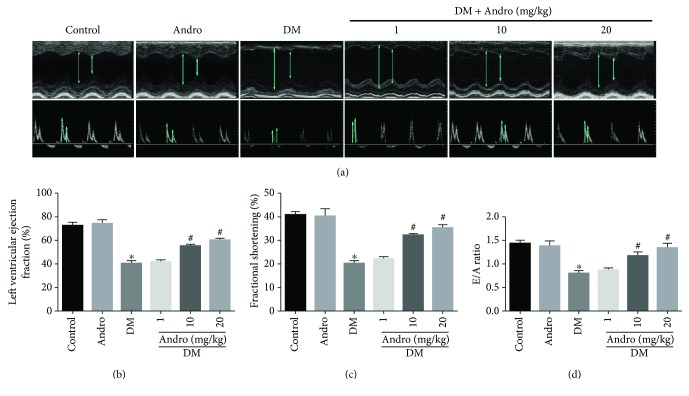
Andro treatment attenuates diabetes-induced myocardial dysfunction *in vivo*. (a) Representative M-mode echocardiograms (upper panel) and representative pulsed-wave Doppler echocardiograms of mitral inflow (lower panel). (b) Left ventricular ejection fraction (LVEF). (c) Fractional shortening (FS). (d) Early to late mitral flow (E/A). Control: normal mice; Andro: normal mice treated with Andro at a dose of 20 mg/kg/day orally by gavage; DM: diabetic mice. ^∗^ *P* < 0.05 compared to control; ^#^ *P* < 0.05 compared to DM.

For the animal experiments, mice were divided into six groups: control, Andro, DM, and DM + Andro (1, 10, and 20 mg/kg). Compared with citrate treatment, STZ induced rapid hyperglycaemia in mice, beginning at one week after injection (Figure S1A). To assess cardiac function, we performed echocardiography and observed a decrease in LVEF, FS, and E/A in diabetic mice compared to control mice (Figures 1(a)–1(d)). Treatment with Andro at both 10 and 20 mg/kg improved the parameters of cardiac function (LVEF, FS, and E/A ratios; Figure 1).

In addition, Andro-treated diabetic mice displayed a significant reduction in blood glucose but an increase in insulin levels, and the effect was dose-dependent (Figure S1B and C). Further, Andro administration (at 10 and 20 mg/kg) reduced mortality in diabetic mice (Figure S1D). These data indicate that the cardioprotective effects afforded by Andro administration are at least in part due to its ability to lower blood glucose levels.

### 3.2. Andro Prevents Diabetes-Induced Myocardial Remodeling

**Figure 2:**
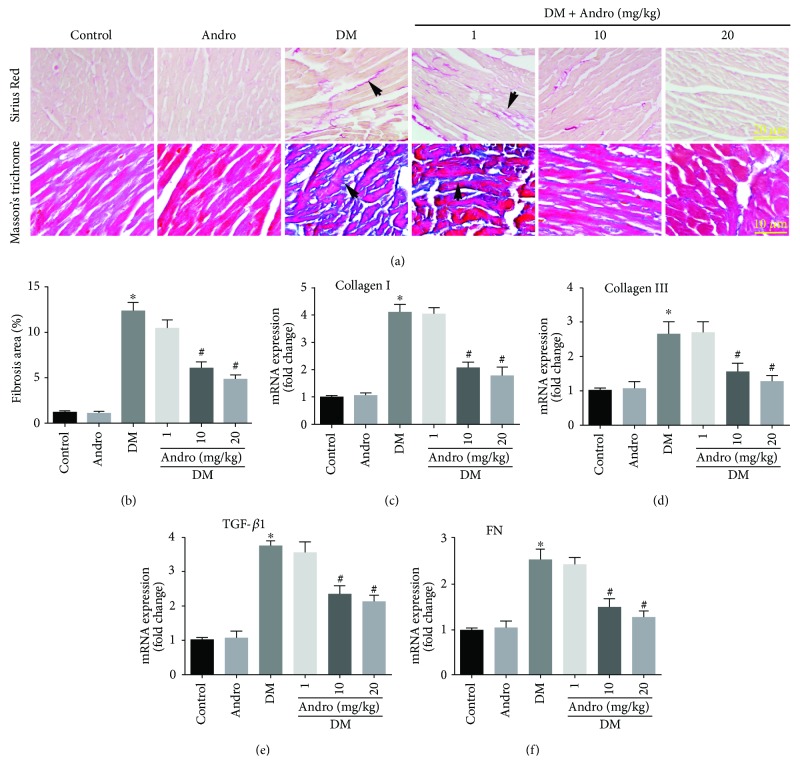
Andro treatment prevents diabetes-induced cardiac fibrosis *in vivo*. (a) Representative Sirius Red staining (upper panel) and Masson's trichrome staining (lower panel). (b) Quantification of Sirius Red-stained collagen in the myocardium. (c–f) Real-time qPCR analysis was performed to detect the mRNA expression of the fibrosis-associated genes, including collagen I, collagen Ш, TGF-*β*1, and FN in the myocardial tissues. ^∗^ *P* < 0.05 compared to control; ^#^ *P* < 0.05 compared to DM.

To evaluate the effect of Andro treatment on diabetes-induced cardiac fibrosis, we performed both Masson's trichrome and Sirius Red staining (Figure 2(a)). Elevated accumulation of collagen was observed in the interstitial regions of diabetic myocardium, as compared to control (Figure 2(a)). Quantification of Sirius Red-stained collagen showed that collagen deposition in the DM + Andro groups (10 and 20 mg/kg) was lower than that in the DM group (Figure 2(b)). We next tested the effect of Andro treatment on fibrotic markers in the diabetic myocardium. The results indicated that the mRNA expression of fibrosis markers including collagen I, collagen Ш, transforming growth factor *β*1 (TGF-*β*1), and fibronectin (FN) was higher in diabetic mice than that in control mice, whereas these changes were attenuated in diabetic mice treated with 10 or 20 mg/kg Andro (Figures 2(c)–2(f)).

**Figure 3:**
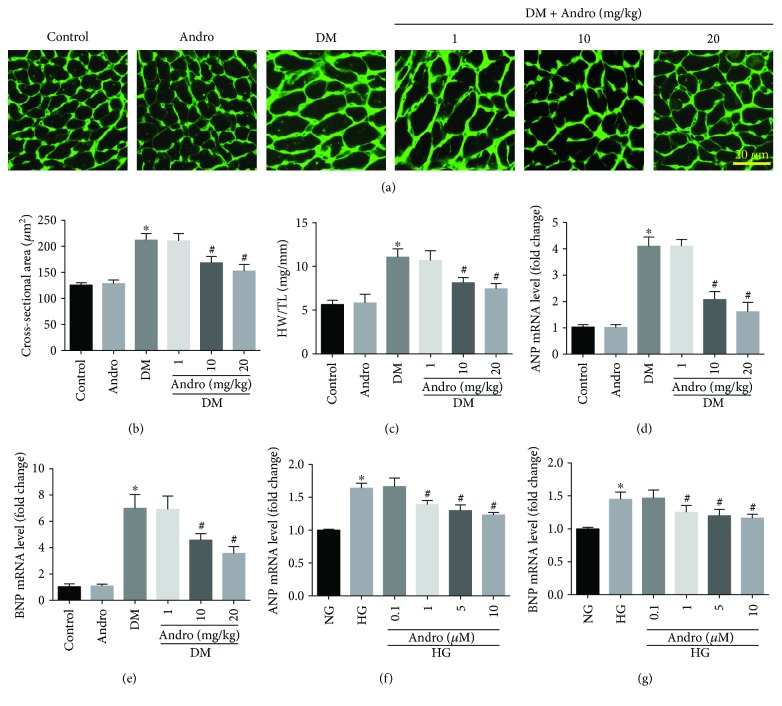
Andro treatment inhibits diabetes-induced cardiac hypertrophy. (a and b) WGA staining showing the cross-sectional area of cardiomyocytes. (c) The ratio of heart weight/tibial length (HW/TL). (d and e) mRNA levels of ANP and BNP in the diabetic myocardium. (f and g) mRNA levels of ANP and BNP in high glucose-stimulated H9c2 cardiomyoblasts. ^∗^ *P* < 0.05 compared to control or NG; ^#^ *P* < 0.05 compared to DM or HG.

Cardiac hypertrophy is also an important feature of DCM. Therefore, the effect of Andro treatment on cardiac hypertrophy was examined. Wheat germ agglutinin (WGA) staining revealed that Andro treatment markedly reduced the increased cross-sectional area of cardiomyocytes in the diabetic myocardium (Figures 3(a) and 3(b)). Andro administration also reduced the increased ratio of heart weight/tibial length (HW/TL) in diabetic mice (Figure 3(c)). Next, we determined the mRNA levels of cardiac hypertrophy marker genes including atrial natriuretic peptide (ANP) and brain natriuretic peptide (BNP) by RT-PCR. The results showed that diabetes-induced ANP and BNP were markedly decreased by Andro administration (Figures 3(d) and 3(e)). These data indicated that Andro treatment attenuated diabetes-induced cardiac hypertrophy. Furthermore, Andro treatment (at 10 and 20 mg/kg) prevented high glucose-induced upregulation of ANP and BNP *in vitro* (Figures 3(f) and 3(g)).

### 3.3. Andro Treatment Mitigates NF-*κ*B-Mediated Inflammatory Response in the Diabetic Myocardium

**Figure 4:**
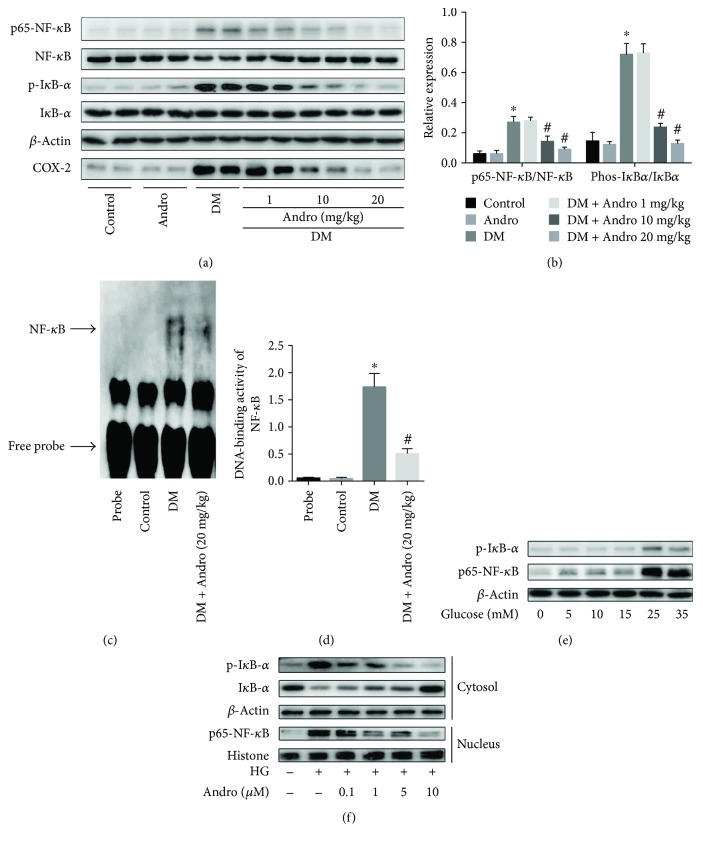
Andro treatment suppresses NF-*κ*B activation in the diabetic myocardium. (a and b) Western blot analysis of p65NF-*κ*B, p-I*κ*B*α*, and COX-2 expression in the myocardial tissues. (c and d) Gel shift assay showed that NF-*κ*B activation was inhibited by Andro treatment. (e) Western blot analysis of p65NF-*κ*B and p-I*κ*B*α* expression in H9c2 cells in response to different glucose concentrations. (f) Effect of Andro treatment on the expression of p65NF-*κ*B and p-I*κ*B*α*. HG, 25 mM glucose. ^∗^ *P* < 0.05 compared to control; ^#^ *P* < 0.05 compared to DM.

A study has shown that high glucose can induce the release of proinflammatory cytokines in a NF-*κ*B-dependent manner in cardiac cells [13]. We then examined the effect of Andro on NF-*κ*B activation in the diabetic myocardium. As shown in Figures 4(a) and 4(b), there was a marked increase in the phosphorylation of I*κ*B*α* and p65NF-*κ*B as well as COX-2, which decreased upon treatment with Andro at 10 and 20 mg/kg. Furthermore, gel shift assay confirmed that Andro treatment inhibited NF-*κ*B activation in the diabetic myocardium (Figures 4(c) and 4(d)).

We next examined the changes of the phospho-NF-*κ*B (p65) and I*κ*B*α* in H9c2 cardiomyoblasts, which were exposed to varying concentrations of glucose (5, 10, 15, 25, or 35 mM) for 48 h. We found that high glucose (HG, 25 and 35 mM) caused significant changes in both phosphorylation of NF-*κ*B (p65) and I*κ*B*α* (Figure 4(e)). Incubation with HG (25 mM) for 48 h caused I*κ*B*α* degradation in the cytosol, with elevated phosphorylation level of I*κ*B*α* inducing the release of active p65 and translocation to the nucleus (Figure 4(f)). As expected, Andro treatment suppressed HG-induced I*κ*B*α* and subsequent active p65 nuclear translocation (Figure 4(f)).

**Figure 5:**
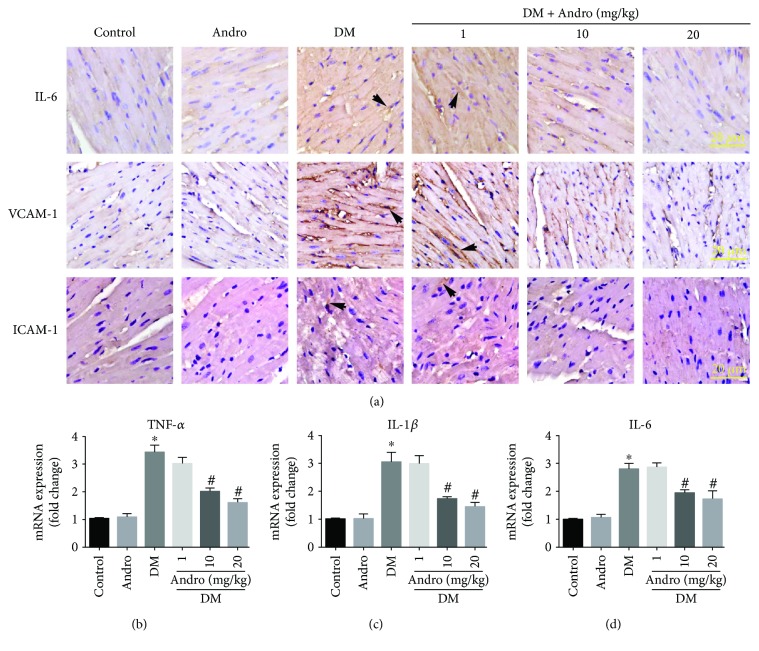
Andro treatment attenuates diabetes-induced myocardial inflammation. (a) Immunostaining of IL-6 (upper panel), VCAM-1 (middle panel), and ICAM-1 (lower panel). (b–d) Real-time qPCR analysis was performed to detect the mRNA expression of the proinflammatory cytokines, including TNF-*α*, IL-1*β*, and IL-6 in the myocardial tissues. ^∗^ *P* < 0.05 compared to control; ^#^ *P* < 0.05 compared to DM.

Furthermore, our data of immunochemistry indicated that Andro treatment (at 10 and 20 mg/kg) inhibited the protein expression of IL-6 and adhesion molecules, including intercellular cell adhesion molecule-1 (ICAM-1) and vascular cell adhesion molecule-1 (VCAM-1) in diabetic myocardial tissues (Figure 5(a)). The mRNA expression of proinflammatory cytokines (TNF-*α*, IL-1*β*, and IL-6) was also hindered by Andro treatment (Figures 5(b)–5(d)).

### 3.4. Andro Treatment Attenuates Diabetes-Induced Myocardial Oxidative and Nitrative Stress

**Figure 6:**
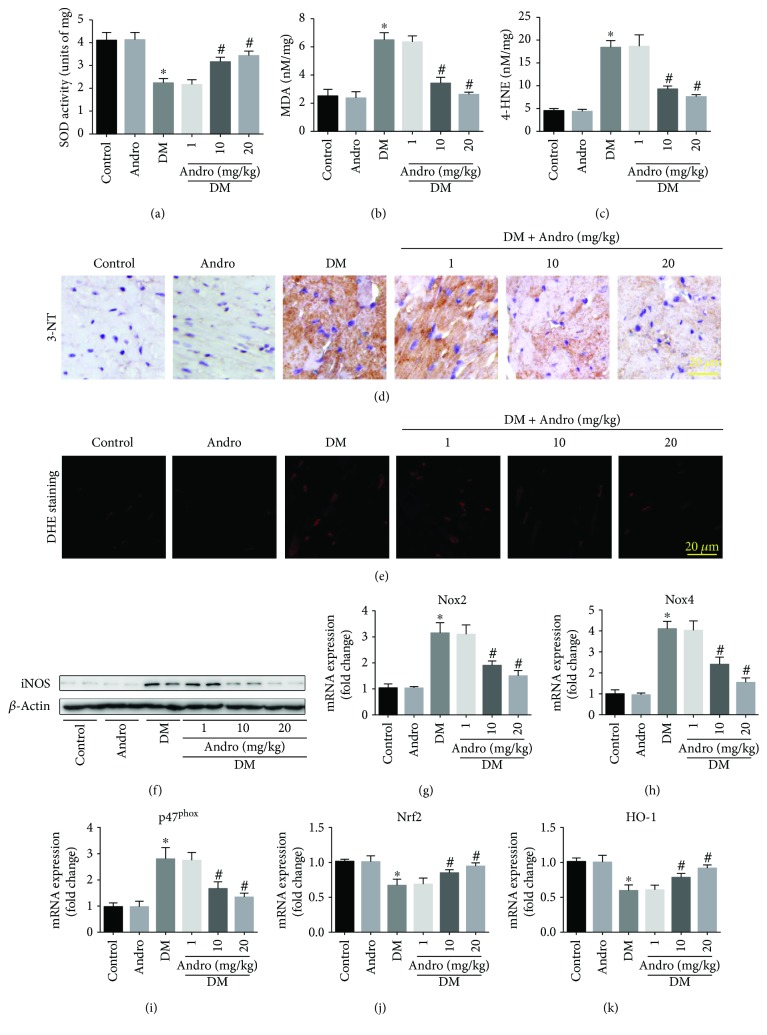
Andro treatment inhibits diabetes-induced myocardial oxidative/nitrative stress. Quantification of SOD (a), MDA (b), and 4-HNE (c) levels in homogenized fresh heart tissues. (d) Immunostaining of 3-NT. (e) Myocardial O_2_^−^ generation was detected by DHE staining in the freshly frozen sections. (f) Western blot analysis of iNOS levels. (g–i) Real-time qPCR analysis was performed to detect the mRNA expression of the ROS-generating NADPH oxidases (NOX2, NOX4, and p47^phox^) in the myocardial tissues. (j and k) The mRNA expression of Nrf2 and HO-1 in the myocardial tissues. ^∗^ *P* < 0.05 compared to control; ^#^ *P* < 0.05 compared to DM.

Oxidative stress drives the development of DCM [9]. We observed markedly reduced SOD activity (Figure 6(a)) along with augmented lipid peroxides (MDA and 4-HNE, Figures 6(b) and 6(c)) in the diabetic myocardium. However, these detrimental changes were attenuated by 12-week treatment with Andro (at 10 and 20 mg/kg) during the course of diabetes. We also found that Andro treatment (at 10 and 20 mg/kg) suppressed the diabetes-induced 3-NT accumulation (Figure 6(d)). We then detected the cardiac O_2_^−^ generation using the fluorescent DHE probe, which revealed that the elevated O_2_^−^ levels in the diabetic myocardium were markedly suppressed by treatment with Andro (Figure 6(e)). Further, Western blot analysis showed that Andro treatment could inhibit the expression of inducible nitric oxide synthase (iNOS) in the diabetic myocardium (Figure 6(f)). Real-time qPCR analysis indicated that Andro remarkably mitigated the expression of various ROS-generating NADPH oxidases (NOX2, NOX4, and p47^phox^) induced by diabetes (Figures 6(g)–6(i)). Treatment with Andro for 12 weeks also reversed the diabetes-induced decrease in Nrf2 and HO-1 levels (Figures 6(j) and 6(k)). These data indicated that Andro treatment could abrogate diabetes-induced myocardial oxidative and nitrative stress.

### 3.5. Andro Treatment Blocks HG-Triggered ROS Generation via Regulating NOXs/Nrf2 Redox Imbalance in H9c2 Cardiomyoblasts

**Figure 7:**
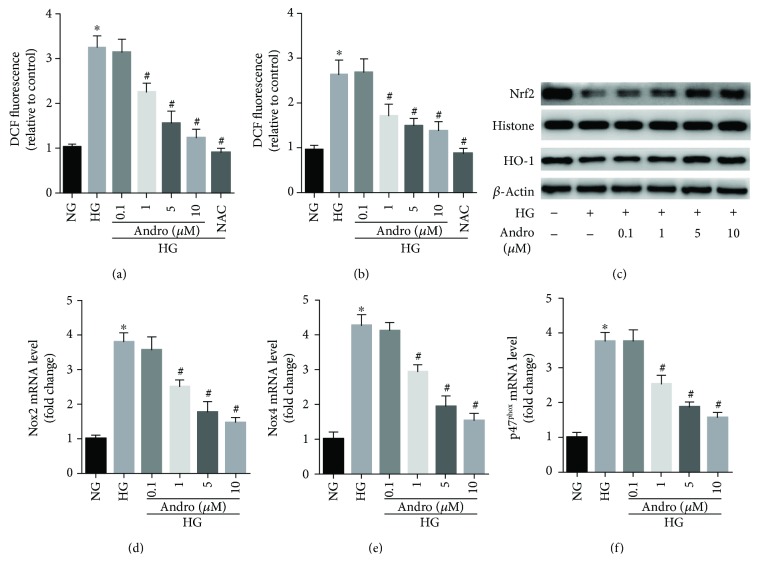
Andro treatment blocks HG-triggered ROS generation via regulating NOX/Nrf2 redox imbalance in H9c2 cardiomyoblasts. (a and b) DCFH-DA and DHE probes were used to measure the levels of cellular ROS using confocal microscopy. The relative fluorescence intensity was acquired. (c) Western blot analysis was performed to evaluate the levels of Nrf2 and HO-1. (d–f) NADPH oxidases (NOX2, NOX4, and p47^phox^) mRNA levels were detected by real-time qPCR analysis. NG, 5.5 mM glucose; HG, 25 mM glucose. ^∗^ *P* < 0.05 compared to NG; ^#^ *P* < 0.05 compared to HG.

We then explored the effect of Andro treatment on HG-triggered ROS generation *in vitro*. Exposure of H9c2 cardiomyoblasts to HG (25 mM) for 48 h induced a relatively rapid generation of ROS, evidenced by elevated intensity of both DCFH-DA and DHE fluorescence (Figures 7(a) and 7(b)). Interestingly, treatment of the H9c2 cells with Andro or NAC hindered the effect of HG on ROS production (Figures 7(a) and 7(b)). As both NOX-driven ROS production and Nrf2-mediated antioxidant response participated in oxidative damage, we wonder whether NOXs/Nrf2 imbalance existed in HG-stimulated H9c2 cardiomyoblasts. Simultaneously, we found that the Nrf2 activity and the expression of the Nrf2-directed heme oxygenase-1 (HO-1) were reduced (Figure 7(c)) while the expression of ROS-generating NADPH oxidases (NOX2, NOX4, and p47^phox^) was significantly increased (Figures 7(d)–7(f)) in HG-stimulated H9c2 cardiomyoblasts. Andro treatment reduced the expression of various NADPH oxidases (NOX2, NOX4, and p47^phox^) but promoted Nrf2 nuclear translocation (Figures 7(c)–7(f)). These results suggest that Andro attenuated HG-triggered ROS generation by inactivating NADPH oxidases and restoring Nrf2/HO-1 expression in H9c2 cardiomyoblasts.

### 3.6. Andro Treatment Attenuates Cell Apoptosis in the Diabetic Myocardium and HG-Stimulated H9c2 Cardiomyoblasts

**Figure 8:**
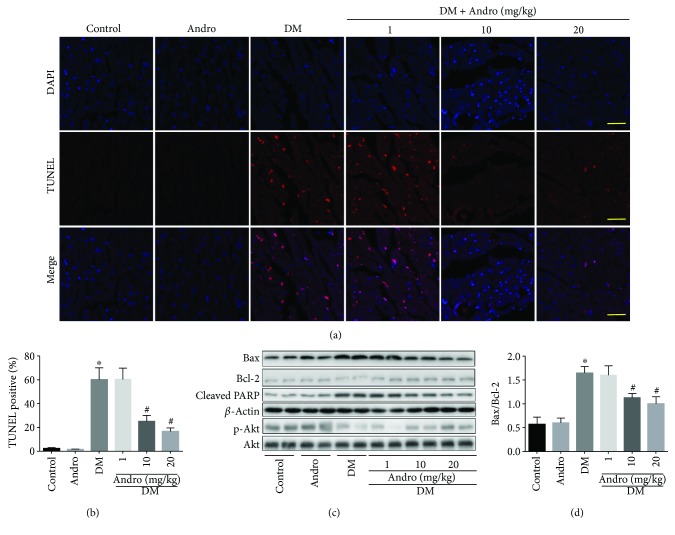
Andro treatment attenuates diabetes-induced myocardial apoptosis. (a) TUNEL-stained sections. Red nuclei indicate apoptotic cells (scale bar = 20 *μ*m). (b) Quantitative analysis of the proportion of TUNEL-positive nuclei. (c) Western blot analysis of p-Akt and cleaved PARP activity, Bax, and Bcl-2. (d) Bax/Bcl-2 ratio. ^∗^ *P* < 0.05 compared to control; ^#^ *P* < 0.05 compared to DM.

In the diabetic myocardium, we observed enhanced apoptosis (Figures 8(a) and 8(b)) and marked increase in Bax/Bcl-2 ratio and cleaved PARP activity (Figures 8(c) and 8(d)). All these detrimental changes were inhibited following Andro treatment (at 10 and 20 mg/kg). In addition, Akt activation was significantly hampered in the diabetic myocardium, and this effect was reversed by Andro treatment (at 10 and 20 mg/kg) (Figure 8(c)). The antiapoptotic activity of Andro is mediated, at least in part, by modulating Akt activity (Figure 8(c)).

**Figure 9:**
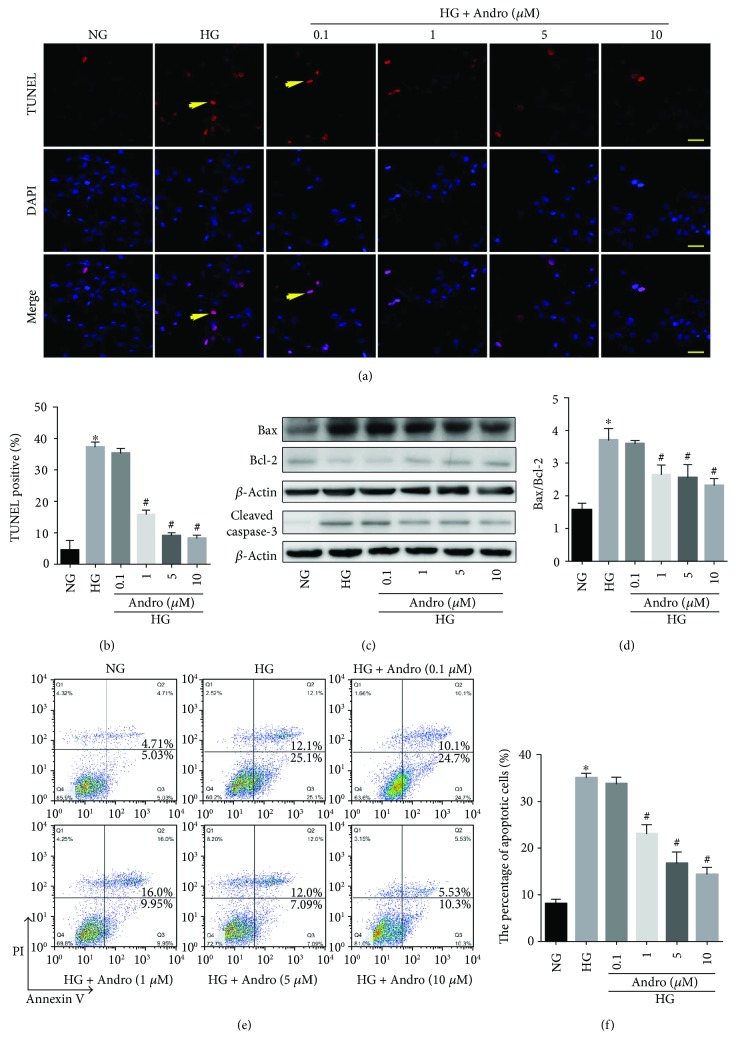
Andro mitigates HG-induced apoptosis in H9c2 cells. (a) TUNEL staining in H9c2 cells (scale bar = 20 *μ*m). (b) Quantitative analysis of the proportion of TUNEL-positive nuclei. (c) Western blot analysis of Bax, Bcl-2, and caspase-3 activity in H9c2 cells. (d) Bax/Bcl-2 ratio in H9c2 cells. (e) Flow cytometry to determine apoptosis in H9c2 cells. (f) Quantitative analysis of apoptotic cells. NG, 5.5 mM glucose; HG, 25 mM glucose. ^∗^ *P* < 0.05 compared to NG; ^#^ *P* < 0.05 compared to HG.

Moreover, we observed a significant increase of apoptosis in H9c2 cardiomyoblasts incubated with HG (25 mM) for 48 h (Figures 9(a) and 9(b)). Andro treatment (1, 5, and 10 *μ*M) markedly attenuated the HG-induced increase in Bax/Bcl-2 ratio and caspase-3 activity (Figures 9(c) and 9(d)). These were further confirmed by flow cytometry (Figures 9(e) and 9(f)).

## 4. Discussion

Our work showed for the first time that Andro treatment dose dependently attenuated cardiac inflammation, oxidative damage, and cardiac apoptosis, which subsequently improved cardiac function in diabetic mice. Consistent with previous studies, diabetic mice exhibited declined myocardial performance, activated NF-*κ*B-mediated inflammation, enhanced oxidative/nitrative stress, and increased cardiac hypertrophy and fibrosis [1, 2, 5, 10–13]. Our results confirmed that treatment with Andro, a natural antibiotic which has been shown to lower blood glucose levels [18] and relieve diabetic nephropathy [21], could abrogate the above detrimental changes and improve cardiac function in diabetic mice. Interestingly, significant dose-dependent effects of Andro were observed. Mechanistically, Andro suppressed NF-*κ*B-mediated inflammation and modulated NOXs/Nrf2-mediated oxidative stress.

Similar to previous reports, NF-*κ*B signaling pathway was activated in both the diabetic myocardium and HG-stimulated cardiac cells [9, 13]. In parallel with NF-*κ*B activation, the levels of NF-*κ*B-directed proinflammatory TNF-*α*, IL-1*β*, and IL-6 were elevated in the diabetic myocardium. Mediators from this inflammatory cascade in turn modulate specific intracellular signaling pathways inducing cardiomyocyte mitochondrial dysfunction and death, fibroblast proliferation, and collagen deposition [9]. In addition, NF-*κ*B activation induces increased oxidative damage and contributes to mitochondrial as well as cardiac dysfunction in diabetes [13]. Maier et al. [30] confirmed that an excessive inflammatory response caused by cardiomyocyte-specific IKK/NF-*κ*B activation was sufficient to induce cardiomyopathy. Thomas et al. [31] demonstrated that cardiomyocyte-specific NF-*κ*B signaling inhibition could attenuate diabetes-induced cardiac dysfunction through the suppression of the cardiac renin-angiotensin system. Interestingly, several anti-inflammatory compounds, such as atorvastatin [32, 33] and cannabidiol [13], were found to be beneficial for the treatment of DCM. Consistent with previous studies [15, 22], our results indicated that Andro treatment effectively suppressed NF-*κ*B activation and the expression of inflammatory cytokines, along with the subsequent decreased myocardial damage in diabetes.

Oxidative damage under hyperglycaemic conditions is an important event in DCM. The hyperglycaemia-induced ROS generation induces lipid peroxidation and thus activates various stress signaling pathways (e.g., NF-*κ*B signaling) [34]. The latter may result in marked nitrative stress, which is also implicated in diabetic complications [13]. It is of interest to note that hyperglycaemia-induced ROS generation activates NF-*κ*B proinflammatory pathway [35] and the nuclear enzyme poly(ADP-ribose) polymerase-1 [8], which in turn increases the levels of proinflammatory cytokines, cell adhesion molecules, and iNOS [8]. Therefore, oxidative/nitrative stress, inflammatory pathways, and stress signaling are closely interrelated and eventually promote DCM development. Thus, targeting just one aspect of pathogenesis seems to be an ineffective approach for treating DCM. Our results showed that Andro treatment could attenuate oxidative/nitrative stress and improve cardiac function. Increasing evidence has indicated that natural agents (including curcumin, resveratrol, and berberine) with antioxidant and antihyperglycemic effects could exert cardioprotective activities [36–38]. As more and more patients have been concerned about the potential adverse effects of chemical or biochemical antidiabetic drugs, natural medicine may be beneficial in attenuating diabetic complications by multitarget therapeutic effects.

Since we have confirmed that Andro protected against oxidative stress in the diabetic hearts, we further demonstrated the protection depended on its capability to restore redox homeostasis in response to hyperglycaemia stimulation. NADPH oxidase complex, composed of membrane-associated NOX homologs (NOX1–NOX5), p22^phox^ subunits, and cytosolic subunits (including p47^phox^, p67^phox^, p40^phox^, and Rac), mediates cardiac oxidative damage in diabetes [11]. Nrf2 is a master cellular sensor for oxidative stress and has been proven to protect the heart from oxidative damage through transcriptionally activating antioxidant genes, including HO-1 and superoxide dismutase [28]. Evidence has indicated that Nrf2 expression was markedly decreased both in the myocardium of STZ-induced diabetic mice and diabetic patients [39]. The balance of NOX-directed ROS production and Nrf2-mediated antioxidant response is involved in oxidative damage. A study by Hecker et al. [40] showed that modulating Nox4-Nrf2 redox imbalance in myofibroblasts was effective to treat age-associated fibrotic disorders. Therefore, restoration of NOXs-Nrf2 redox balance has the potential to alleviate DCM or even reverse its progression. We found the expression of NOX2, NOX4, and p47^phox^ was elevated while the expression of the Nrf2-directed HO-1 expression was decreased, suggesting that NOXs/Nrf2 imbalance existed in both the diabetic myocardium and HG-stimulated H9c2 cardiomyoblasts. Andro treatment reduced the expression of NADPH oxidases, promoted Nrf2 nuclear translocation, and thus restored NOXs/Nrf2 redox balance. This was consistent with the finding of Lin et al. [24], who observed that Andro could activate Nrf2/HO-1 axis and thus inhibit hypoxia-induced ET-1 secretion in endothelial cells. In addition, Akt activation was significantly hampered but reversed by Andro treatment in the diabetic myocardium. The antiapoptotic activity of Andro is mediated, at least in part, by its ability to modulate Akt activity. Further, evidence has indicated that PI3K/Akt is also essential for Nrf2 activation [41]. It may provide a clue for how Andro mediates Nrf2 nuclear translocation.

## 5. Conclusions

Diabetes-induced cardiac hypertrophy, fibrosis, and cardiac dysfunction were attenuated by Andro treatment. Our results reveal that Andro exerts cardioprotection in diabetic mice by regulating NOXs/Nrf2-mediated oxidative stress and NF-*κ*B-mediated inflammation and apoptosis. Our study provides scientific evidence that Andro may be a promising compound for the treatment of DCM.

## Data Availability

All data that support the findings of this study are included in this article (and its supplementary information files). If the readers need further datasets analyzed during the current study, it is available from the corresponding author upon reasonable request.

## Supplementary Materials

Supplementary Materials Supplemental Figure 1: measurement of blood glucose and insulin levels and the mortality of the mice.

## References

[1] Feng B., Chen S., George B., Feng Q., Chakrabarti S. (2010). miR133a regulates cardiomyocyte hypertrophy in diabetes. *Diabetes/Metabolism Research and Reviews*.

[2] Kajstura J., Fiordaliso F., Andreoli A. M. (2001). IGF-1 overexpression inhibits the development of diabetic cardiomyopathy and angiotensin II–mediated oxidative stress. *Diabetes*.

[3] Van Linthout S., Spillmann F., Riad A. (2008). Human apolipoprotein A-I gene transfer reduces the development of experimental diabetic cardiomyopathy. *Circulation*.

[4] Gilbert R. E., Krum H. (2015). Heart failure in diabetes: effects of anti-hyperglycaemic drug therapy. *The Lancet*.

[5] Al-Rasheed N. M., Al-Rasheed N. M., Hasan I. H. (2017). Simvastatin ameliorates diabetic cardiomyopathy by attenuating oxidative stress and inflammation in rats. *Oxidative Medicine and Cellular Longevity*.

[6] Varga Z. V., Giricz Z., Liaudet L., Hasko G., Ferdinandy P., Pacher P. (2015). Interplay of oxidative, nitrosative/nitrative stress, inflammation, cell death and autophagy in diabetic cardiomyopathy. *Biochimica et Biophysica Acta (BBA) - Molecular Basis of Disease*.

[7] Berthiaume J. M., Kurdys J. G., Muntean D. M., Rosca M. G. (2017). Mitochondrial NAD^+^/NADH redox state and diabetic cardiomyopathy. *Antioxidants & Redox Signaling*.

[8] Qin W. D., Liu G. L., Wang J. (2016). Poly(ADP-ribose) polymerase 1 inhibition protects cardiomyocytes from inflammation and apoptosis in diabetic cardiomyopathy. *Oncotarget*.

[9] Zhong P., Wu L., Qian Y. (2015). Blockage of ROS and NF-*κ*B-mediated inflammation by a new chalcone L6H9 protects cardiomyocytes from hyperglycemia-induced injuries. *Biochimica et Biophysica Acta (BBA) - Molecular Basis of Disease*.

[10] Pan Y., Wang Y., Zhao Y. (2014). Inhibition of JNK phosphorylation by a novel curcumin analog prevents high glucose–induced inflammation and apoptosis in cardiomyocytes and the development of diabetic cardiomyopathy. *Diabetes*.

[11] Hansen S. S., Aasum E., Hafstad A. D. (2018). The role of NADPH oxidases in diabetic cardiomyopathy. *Biochimica et Biophysica Acta (BBA) - Molecular Basis of Disease*.

[12] Frati G., Schirone L., Chimenti I. (2017). An overview of the inflammatory signalling mechanisms in the myocardium underlying the development of diabetic cardiomyopathy. *Cardiovascular Research*.

[13] Rajesh M., Mukhopadhyay P., Bátkai S. (2010). Cannabidiol attenuates cardiac dysfunction, oxidative stress, fibrosis, and inflammatory and cell death signaling pathways in diabetic cardiomyopathy. *Journal of the American College of Cardiology*.

[14] Cabrera D., Wree A., Povero D. (2017). Andrographolide ameliorates inflammation and Fibrogenesis and attenuates Inflammasome activation in experimental non-alcoholic steatohepatitis. *Scientific Reports*.

[15] Nie X., Chen S.-R., Wang K. (2017). Attenuation of innate immunity by andrographolide derivatives through NF-*κ*B signaling pathway. *Scientific Reports*.

[16] Ding Y., Chen L., Wu W., Yang J., Yang Z., Liu S. (2017). Andrographolide inhibits influenza a virus-induced inflammation in a murine model through NF-*κ*B and JAK-STAT signaling pathway. *Microbes and Infection*.

[17] Mishra S. K., Tripathi S., Shukla A., Oh S. H., Kim H. M. (2015). Andrographolide and analogues in cancer prevention. *Frontiers in Bioscience (Elite edition)*.

[18] Yu B. C., Hung C. R., Chen W. C., Cheng J. T. (2003). Antihyperglycemic effect of andrographolide in streptozotocin-induced diabetic rats. *Planta Medica*.

[19] Yu B. C., Chang C. K., Su C. F., Cheng J. T. (2008). Mediation of *β*-endorphin in andrographolide-induced plasma glucose-lowering action in type I diabetes-like animals. *Naunyn-Schmiedeberg's Archives of Pharmacology*.

[20] Chern C. M., Liou K. T., Wang Y. H., Liao J. F., Yen J. C., Shen Y. C. (2011). Andrographolide inhibits PI3K/AKT-dependent NOX2 and iNOS expression protecting mice against hypoxia/ischemia-induced oxidative brain injury. *Planta Medica*.

[21] Ji X., Li C., Ou Y. (2016). Andrographolide ameliorates diabetic nephropathy by attenuating hyperglycemia-mediated renal oxidative stress and inflammation via Akt/NF-*κ*B pathway. *Molecular and Cellular Endocrinology*.

[22] Yu Z., Lu B., Sheng Y., Zhou L., Ji L., Wang Z. (2015). Andrographolide ameliorates diabetic retinopathy by inhibiting retinal angiogenesis and inflammation. *Biochimica et Biophysica Acta (BBA) - General Subjects*.

[23] Lan T., Wu T., Gou H. (2013). Andrographolide suppresses high glucose-induced fibronectin expression in mesangial cells via inhibiting the AP-1 pathway. *Journal of Cellular Biochemistry*.

[24] Lin H.-C., Su S.-L., Lu C.-Y. (2017). Andrographolide inhibits hypoxia-induced HIF-1*α*-driven endothelin 1 secretion by activating Nrf2/HO-1 and promoting the expression of prolyl hydroxylases 2/3 in human endothelial cells. *Environmental Toxicology*.

[25] Rajesh M., Batkai S., Kechrid M. (2012). Cannabinoid 1 receptor promotes cardiac dysfunction, oxidative stress, inflammation, and fibrosis in diabetic cardiomyopathy. *Diabetes*.

[26] Suo X. B., Zhang H., Wang Y. Q. (2007). HPLC determination of andrographolide in rat whole blood: study on the pharmacokinetics of andrographolide incorporated in liposomes and tablets. *Biomedical Chromatography*.

[27] Liu X., Song X., Lu J. (2016). Neferine inhibits proliferation and collagen synthesis induced by high glucose in cardiac fibroblasts and reduces cardiac fibrosis in diabetic mice. *Oncotarget*.

[28] Dassano A., Mancuso M., Giardullo P. (2014). N^6^-isopentenyladenosine and analogs activate the NRF2-mediated antioxidant response. *Redox Biology*.

[29] Liang E. S., Cheng W., Yang R. X., Bai W. W., Liu X., Zhao Y. X. (2018). Peptidyl-prolyl isomerase Pin1 deficiency attenuates angiotensin II-induced abdominal aortic aneurysm formation in ApoE^−/−^ mice. *Journal of Molecular and Cellular Cardiology*.

[30] Maier H. J., Schips T. G., Wietelmann A. (2012). Cardiomyocyte-specific I*κ*B kinase (IKK)/NF-*κ*B activation induces reversible inflammatory cardiomyopathy and heart failure. *Proceedings of the National Academy of Sciences of the United States of America*.

[31] Thomas C. M., Yong Q. C., Rosa R. M. (2014). Cardiac-specific suppression of NF-*κ*B signaling prevents diabetic cardiomyopathy via inhibition of the renin-angiotensin system. *American Journal of Physiology-Heart and Circulatory Physiology*.

[32] Van Linthout S., Riad A., Dhayat N. (2007). Anti-inflammatory effects of atorvastatin improve left ventricular function in experimental diabetic cardiomyopathy. *Diabetologia*.

[33] Ren X.-M., Zuo G.-F., Wu W. (2016). Atorvastatin alleviates experimental diabetic cardiomyopathy by regulating the GSK-3*β*-PP2Ac-NF-*κ*B signaling axis. *PLoS One*.

[34] Geraldes P., King G. L. (2010). Activation of protein kinase C isoforms and its impact on diabetic complications. *Circulation Research*.

[35] Huang Z., Zhuang X., Xie C. (2016). Exogenous hydrogen sulfide attenuates high glucose-induced cardiotoxicity by inhibiting NLRP3 Inflammasome activation by suppressing TLR4/NF-*κ*B pathway in H9c2 cells. *Cellular Physiology and Biochemistry*.

[36] Yu W., Wu J., Cai F. (2012). Curcumin alleviates diabetic cardiomyopathy in experimental diabetic rats. *PLoS One*.

[37] Gao Y., Kang L., Li C. (2016). Resveratrol ameliorates diabetes-induced cardiac dysfunction through AT1R-ERK/p38 MAPK signaling pathway. *Cardiovascular Toxicology*.

[38] Chang W., Zhang M., Meng Z. (2015). Berberine treatment prevents cardiac dysfunction and remodeling through activation of 5′-adenosine monophosphate-activated protein kinase in type 2 diabetic rats and in palmitate-induced hypertrophic H9c2 cells. *European Journal of Pharmacology*.

[39] Tan Y., Ichikawa T., Li J. (2011). Diabetic downregulation of Nrf2 activity via ERK contributes to oxidative stress–induced insulin resistance in cardiac cells in vitro and in vivo. *Diabetes*.

[40] Hecker L., Logsdon N. J., Kurundkar D. (2014). Reversal of persistent fibrosis in aging by targeting Nox4-Nrf2 redox imbalance. *Science Translational Medicine*.

[41] Han D., Chen W., Gu X. (2017). Cytoprotective effect of chlorogenic acid against hydrogen peroxide-induced oxidative stress in MC3T3-E1 cells through PI3K/Akt-mediated Nrf2/HO-1 signaling pathway. *Oncotarget*.

