# Supplementary figures and images for: Andrographolide Ameliorates Diabetic Cardiomyopathy in Mice by Blockage of Oxidative Damage and NF-κB-Mediated Inflammation
Source: Oxid Med Cell Longev. 2018 Jun 25;2018:9086747. doi: 10.1155/2018/9086747 (PMC6036810; doi:10.1155/2018/9086747)

**A**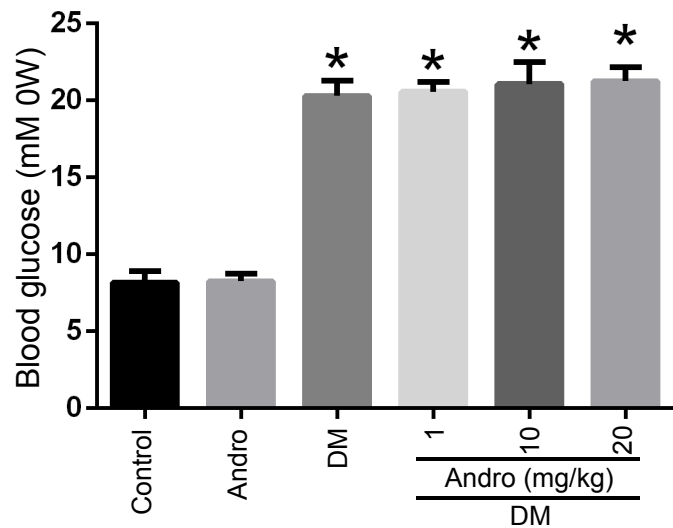**B**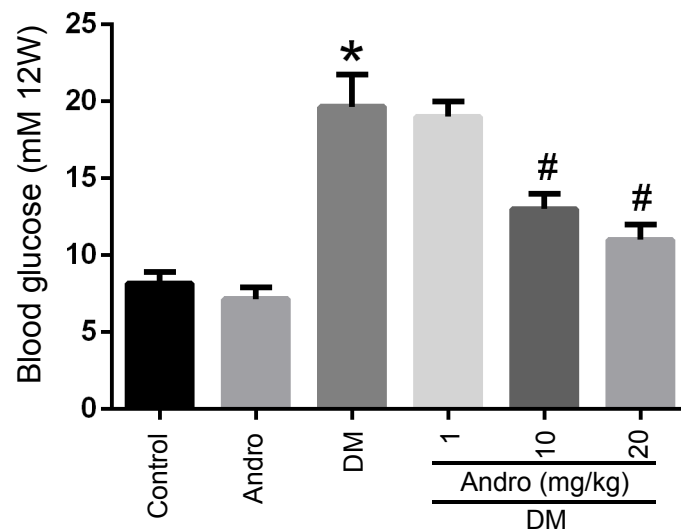**C**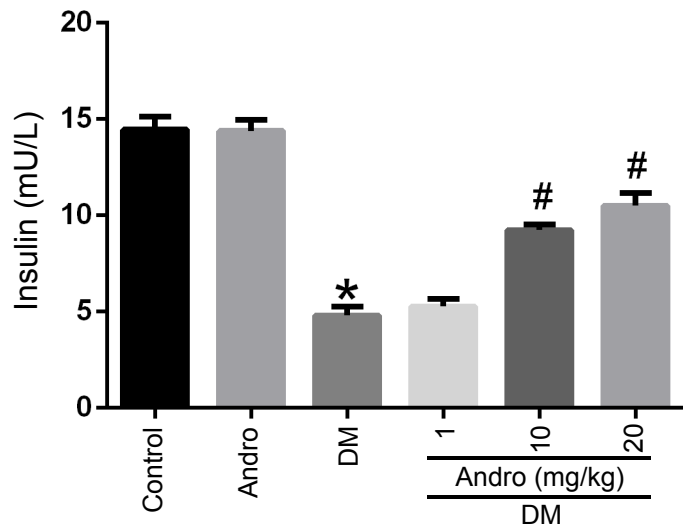**D**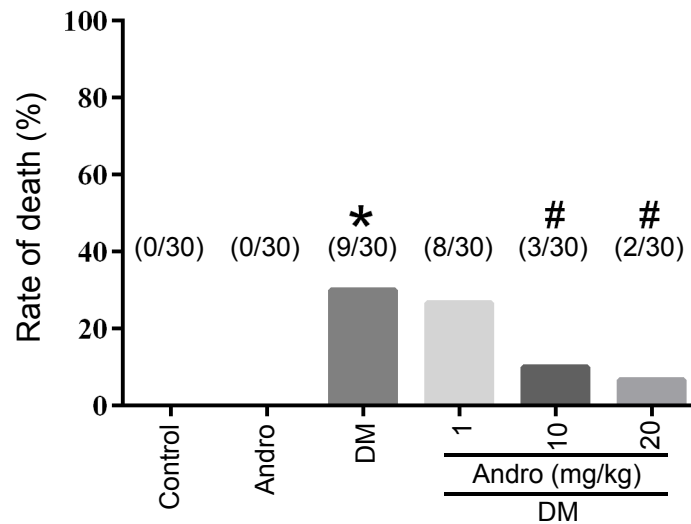

Supplement: Supplementary Materials — Supplemental Figure 1: measurement of blood glucose and insulin levels and the mortality of the mice. [file 9086747.f1.pdf]
